# Antioxidants in male infertility: from empirical supplementation to phenotype directed therapy

**DOI:** 10.3389/fendo.2026.1971018

**Published:** 2026-10-13

**Authors:** Mohammad Ishraq Zafar, Mengyuan Qu

**Affiliations:** 1 School of Health and Nursing, Wuchang University of Technology, Wuhan, China; 2 The Reproduction Medical Center, Shenzhen Luohu People’s Hospital, Shantou University Medical College, Shenzhen, Guangdong, China; 3 The Third Affiliated Hospital of Shenzhen University, Shenzhen University, Shenzhen, Guangdong, China

**Keywords:** antioxidant supplementation, antioxidants, male infertility, redox modulating intervention, therapeutics

## Abstract

Oxidative stress is a biologically plausible contributor to male infertility, and antioxidant supplementation has been investigated for decades. Yet clinical evidence has not converged on a reproducible benefit in reproductive outcomes. The 2025 World Health Organization (WHO) infertility guideline made no recommendation for or against antioxidant supplementation in men with infertility and one or more semen parameters outside WHO reference ranges, reflecting uncertainty rather than proof of universal inefficacy. A Cochrane review identified 90 randomized controlled trials involving 10,303 men, but live birth was reported in only 12 trials with very low certainty. Contemporary randomized trials have not resolved the uncertainty: MOXI found no improvement in semen parameters, sperm DNA fragmentation, pregnancy, or live birth with a multicomponent antioxidant formulation; FAZST found no benefit of folic acid and zinc on semen quality or live birth with an increase in sperm DNA fragmentation; and SUMMER found no improvement in ongoing pregnancy with a combined antioxidant supplement. These findings make routine empirical supplementation difficult to justify but do not establish universal inefficacy of redox modulating interventions. Most trials have enrolled men according to clinical infertility or semen criteria rather than a prospectively defined standardized redox phenotype and have rarely tested treatment effect modification. Biological and clinical heterogeneity, differences among interventions, physiological roles of reactive oxygen species, and reliance on surrogate endpoints may dilute true effects. Future trials should determine which biologically defined men, if any, benefit from which redox modulating intervention, using standardized phenotyping, target engagement measures, prespecified treatment by phenotype interaction testing, and clinically meaningful reproductive outcomes.

## The clinical problem

Male infertility is not a single disease. Causes include impaired spermatogenesis, endocrine and genetic disorders, obstruction, varicocele, genital tract infection or inflammation, metabolic dysfunction, environmental exposures, and conditions in which no single cause is identified (1). This heterogeneity is central to interpreting antioxidant trials: two men with similar semen parameters may have very different upstream mechanisms.

Semen analysis remains the cornerstone of laboratory evaluation, but individual semen parameters show substantial biological variability and only imperfectly predict reproductive potential (2, 3). The WHO sixth edition laboratory manual emphasizes standardized collection, examination, quality control, and reporting (4). Consequently, modest changes in concentration, motility, or morphology should not automatically be interpreted as evidence of improved fertility.

The biological rationale for antioxidant therapy is nevertheless substantial. Spermatozoa contain polyunsaturated fatty acids and have limited cytoplasmic antioxidant defenses; excessive oxidation can damage lipids, proteins, mitochondrial function, and nuclear or mitochondrial DNA (5–8). Oxidative stress has been implicated in several infertility associated conditions, including varicocele, obesity and metabolic dysfunction, inflammation, smoking, and environmental exposures (9–12).

The therapeutic inference is that reducing pathological oxidation could restore sperm function. However, reactive oxygen species (ROS) are not intrinsically harmful. Controlled ROS generation participates in sperm capacitation, hyperactivation, and other physiological signaling processes (5, 13). The relevant therapeutic target is therefore redox homeostasis rather than maximal ROS suppression. This distinction is frequently lost when heterogeneous compounds are grouped under the single label “*antioxidant*”.

## The WHO guideline creates a defining evidence gap

In 2025, the WHO published its first global guideline for the prevention, diagnosis, and treatment of infertility, including recommendations addressing infertility due to male factors (14–16). For men with infertility and one or more semen parameters outside WHO reference ranges who are attempting pregnancy with or without medically assisted reproduction, the Guideline Development Group did not recommend for or against antioxidant supplements (15, 16). The accompanying evidence to decision material identifies important uncertainty and research needs (15).

A commentary published online in January 2026 by Esteves, De Jonge, Pacey, and Vazquez-Levin emphasized that the guideline provides a practical evidence based framework while underscoring major diagnostic and therapeutic gaps in male infertility (17). The antioxidant question is therefore no longer simply whether another supplement should be tested. It is why a field with decades of trials has not generated actionable evidence, and what trial architecture could resolve that uncertainty.

The evidentiary landscape has also become more heterogeneous at the guideline level. A 2026 Global Andrology Forum (GAF) clinical guideline provides recommendations supporting selective antioxidant use in several clinical contexts, including idiopathic infertility, elevated sperm DNA fragmentation, and recognized risk factors for oxidative stress, while acknowledging substantial variation by antioxidant agent, regimen, and patient population (18). These recommendations were developed through a Delphi process followed by expert GRADE assessment and therefore differ methodologically from the WHO guideline’s evidence to decision framework. The divergence is informative: it illustrates how expert consensus may support selective clinical use even when randomized evidence remains insufficient to support a universal recommendation.

## A large literature, but limited clinical convergence

The 2022 Cochrane review included 90 RCTs and 10,303 subfertile men across multiple antioxidant interventions (19). A possible increase in live birth was observed in the pooled analysis, but the certainty was rated very low; only 12 trials reported live birth, and the apparent benefit was no longer statistically significant after exclusion of trials at high risk of bias (19). The correct inference is therefore uncertainty, not demonstrated efficacy. Key evidence is summarized in **Table 1**.

**Table 1:** Selected evidence that defines the current uncertainty in antioxidant therapy for male infertility.

Evidence stream	Participation/design	Treatment/primary endpoint	Treatment effect	Limitation/interpretation
Cochrane review (2022) (19)	90 RCTs; n=10,303	The analysis included a range of interventions, comprising antioxidants (carotenoids, coenzyme Q10, cysteines, melatonin, resveratrol, vitamin C, and vitamin E), antioxidant cofactors (selenium, zinc, and magnesium), and other metabolic nutrients (arginine, carnitines, folic acid, B vitamins, vitamin D with calcium, and polyunsaturated fatty acids). Live birth reported in 12 trials	Possible live birth signal, very low certainty evidence; signal no longer statistically significant after exclusion of high risk of bias trials	Broad heterogeneous evidence base; certainty remains very low
MOXI (2020) (20)	Randomized; Multicenter, Double blind, placebo controlled RCT with an internal pilot study, (n=171)	Vitamin C, vitamin E, vitamin D, selenium, L-carnitine, zinc, folic acid, and lycopene; 3–6 months; primary: live birth; internal pilot endpoints: semen parameters and SDF	Live birth 15% vs 24%; P = 0.14; 95% CI not reported	Recruitment stopped after the internal pilot because prespecified pilot criteria were not met; live birth analysis was not powered for definitive efficacy
MOXI biomarker analysis (2021) (21)	Secondary analysis study of MOXI clinical study; baseline plasma vitamin E (n=131), selenium (n=124), zinc (n=128)	Baseline concentrations; clinical outcomes assessed through trial follow-up	Vitamin E, selenium and zinc concentrations were not associated with pregnancy or live birth in the studied population; Measured baseline concentrations were within normal ranges	Association/prognostic analysis; not a formal test of treatment by biomarker interaction
FAZST (2020) (22)	Randomized, multicenter, double blind, placebo controlled RCT; n= 2,370	Folic acid 5 mg + elemental zinc 30 mg daily; 6 months; co-primary endpoints: live birth resulting from pregnancies occurring within 9 months of randomization, and semen quality at 6 months after randomization	Live birth 34% vs 35%; adjusted RD −0.9% (95% CI −4.7% to 2.8%); adjusted RR 0.98 (95% CI 0.88–1.09). SDF: mean difference +2.4% (95% CI 0.5%–4.4%)	75% attended the final 6 month visit; no benefit for primary outcomes, with higher SDF in the supplement group
SUMMER (2025) (23)	n=1,217 patients were randomized; n=1,171 were included in the primary data	Impryl daily; 6 months; primary ongoing pregnancy rate within 6 months	Primary: 33.8% vs 37.5%; adjusted OR 0.85 (95% CI 0.66–1.09), P = 0.20. Secondary 4–6 month window: 15.5% vs 21.5%; adjusted OR 0.66 (0.47–0.94), P = 0.02	Primary outcome neutral; 4–6 month finding was secondary and should not be conflated with the primary result
WHO guideline (2025) (14–16)	Evidence to decision assessment	Not applicable; Guideline recommendation	No recommendation for or against antioxidant supplementation	Evidence insufficient for routine clinical recommendation

Effect estimates are reported as published. Evidence citations are shown in parentheses. The table summarizes the principal evidence streams and is not a formal ranking of individual antioxidant agents.

MOXI, Males, Antioxidants, and Infertility; RCT, randomized controlled trial; SDF, sperm DNA fragmentation; WHO, World Health Organization.

Earlier systematic reviews similarly reported heterogeneity in antioxidant type, dose, treatment duration, population, and outcome definition (24, 25). Network meta-analysis has suggested that some agents may improve selected semen parameters, but these analyses remain primarily hypothesis generating when clinical pregnancy and live birth are sparse or inconsistently reported (26). A 2026 Global Andrology Forum clinical guideline, developed through structured international expert consensus, represents the field’s most recent attempt to translate this evidence base into clinical guidance and provides a natural point of comparison for the framework proposed here (18).

The MOXI trial was a multicenter, double blind, placebo controlled RCT conducted at nine US sites (20). The trial tested a combination containing vitamin C, vitamin E, vitamin D, selenium, L-carnitine, zinc, folic acid, and lycopene. The antioxidant group did not show improvement in semen parameters or sperm DNA fragmentation, and pregnancy and live birth outcomes did not support a clinically meaningful benefit (20). A secondary analysis found that baseline plasma vitamin E, selenium, and zinc concentrations were not associated with pregnancy or live birth in the studied population; measured baseline concentrations were within normal ranges. This analysis provides information on baseline association, not evidence for or against treatment effect modification, because a formal treatment by biomarker interaction was not evaluated in the analysis (21).

A separate large multicenter trial randomized 2,370 men seeking infertility treatment to daily folic acid and zinc supplementation or placebo. Supplementation did not improve semen quality parameters or live birth and was associated with a modest increase in sperm DNA fragmentation compared with placebo (22). Folic acid and zinc are only two of the many compounds marketed as antioxidants, but this trial remains one of the largest randomized trials evaluating micronutrient supplementation in this setting and adds to the evidence that empirical micronutrient supplementation has not produced consistent clinical benefit in unselected populations.

The SUMMER trial provides one of the largest contemporary tests of empirical multicomponent supplementation (23). In 1,171 men seeking fertility care, ongoing pregnancy did not differ significantly between antioxidant and placebo groups overall. The primary report concluded that the tested supplement did not improve pregnancy outcomes and did not support its use in this population. The trial therefore provides evidence against routine use of that particular multicomponent strategy in an unselected fertility care population; it does not establish that every antioxidant compound, dose, duration, or biologically selected subgroup is ineffective.

## Why have trials failed to converge?

### Heterogeneity is not statistical noise; it may be the biology

A conventional RCT estimates an average treatment effect. If only a subset of participants has a causal redox abnormality that is modifiable by the intervention, an average treatment effect can approach zero even when a clinically important subgroup benefit exists. Conversely, if redox imbalance is merely downstream of an untreated primary disorder, antioxidant treatment may change a biomarker without sufficiently modifying the causal pathway to improve fertility.

### “Antioxidant” is not a pharmacological class

Antioxidant interventions are heterogeneous in composition, formulation, dose, and biological effects (24–26). For example, clinical studies of CoQ10 have reported effects on semen parameters and oxidative stress related measures (27–29). Pooling these findings conceptually can obscure agent specific effects. A negative trial of a multicomponent formulation should not be generalized to every redox modulating intervention.

### Redox biology is bidirectional

Physiological ROS signaling is required for sperm maturation and fertilization related processes (5, 13). Therefore, the therapeutic objective should be normalization of pathological oxidative burden rather than indiscriminate ROS elimination. This creates a plausible basis for a potentially non-linear relationship between baseline oxidative state and treatment response and provides a mechanistic reason why empiric treatment of unselected populations may fail.

### Oxidative stress may be causal, mediating, or epiphenomenal

Obesity and metabolic dysfunction can alter inflammation, mitochondrial function, endocrine signaling, and redox balance (9, 10). Varicocele can produce hyperthermia, altered venous physiology, hypoxia, and oxidative stress (30). Inflammatory leukocytes can generate ROS (11). If the dominant upstream driver remains untreated, antioxidant therapy may have limited ability to change reproductive outcomes.

### Surrogate outcomes dominate the literature

Sperm concentration, motility, morphology, ROS, and DNA fragmentation are biologically informative, but none is equivalent to live birth (2, 19, 31, 32). Improvement in surrogate endpoints has sometimes been interpreted as evidence of improved fertility, despite their imperfect relationship with live birth. This is particularly problematic when trials are small and underpowered for pregnancy or live birth.

An additional problem is compartmentalization. Oxidative biology measured in ejaculated semen is not necessarily equivalent to oxidative biology within the testis, epididymis, or individual sperm subpopulations. Ejaculated semen contains leukocytes and spermatozoa, each of which can contribute to measured ROS (11, 33). Consequently, a single bulk semen assay may identify a biochemical state without identifying the cellular source, anatomical origin, or causal pathway of the signal. This distinction is particularly important when the proposed intervention acts systemically but the presumed therapeutic target is localized to sperm or the male reproductive tract.

Assay heterogeneity further complicates phenotype definition. Measures such as chemiluminescent ROS, oxidation reduction potential (ORP), total antioxidant capacity, lipid peroxidation products, oxidative sperm DNA-base damage, including 8-hydroxy-2’-deoxyguanosine (8-OHdG) (34, 35), and sperm DNA fragmentation assays quantify related but nonidentical biological processes (31, 32, 36). Preanalytical factors including abstinence interval, time from ejaculation to analysis, temperature, semen liquefaction, and cryopreservation can influence measurements (4). A future enrichment strategy should therefore specify the biological construct being measured, the analytical platform, quality control procedures, timing of sampling, and a clinically or experimentally justified threshold. A biomarker should not be promoted from association to treatment selection criterion merely because it correlates with an adverse reproductive outcome.

Baseline nutritional status may also contribute to treatment effect heterogeneity. An intervention designed to correct a micronutrient deficiency is conceptually different from pharmacologically suppressing oxidative signaling in a nutritionally replete individual. The same nominal dose may therefore represent replacement in one patient and excess exposure in another. These considerations can complicate mechanistic interpretation of multinutrient trials; a null result could reflect failure of the formulation, inappropriate target selection, inadequate exposure, insufficient duration, or absence of a modifiable deficiency rather than failure of the underlying biological pathway.

Duration of treatment also deserves explicit consideration. Human spermatogenesis takes approximately 70–75 days (37), followed by additional time for epididymal maturation; therefore, trials evaluating sperm outcomes after very short exposure may be biologically underpowered even when the nominal sample size is adequate. Conversely, extending treatment does not guarantee benefit if the intervention does not modify the causal pathway. The relevant design question is not simply whether treatment lasts three or six months, but whether the pharmacodynamic effect is expected to occur within the interval during which the prespecified endpoint is assessed.

Finally, infertility trials must recognize that pregnancy is a couple level outcome. Female partner age and reproductive status, tubal factors, treatment allocation in assisted reproduction, coital exposure, and changes in fertility management can influence pregnancy independently of the male intervention (19, 20). These variables do not invalidate trials of male-directed interventions; rather, they require careful eligibility criteria, stratification or covariate adjustment where appropriate, prespecified estimands, and transparent reporting. The strongest efficacy trials should make clear whether the intervention is intended to improve natural conception, improve outcomes during IUI or IVF/ICSI, or improve a specific sperm mediated endpoint. Mixing these clinical contexts without a coherent causal framework can obscure an otherwise meaningful treatment effect.

## The critical gap: predictive phenotyping

The field should distinguish prognostic from predictive biomarkers. A prognostic biomarker is associated with outcome irrespective of treatment. A predictive biomarker identifies patients whose treatment effect differs according to biomarker status. The latter requires a formal treatment by biomarker interaction, not merely a statistically significant treatment effect in one subgroup and a nonsignificant effect in another (38).

In this review, a redox phenotype refers to a prespecified biological state characterized by an appropriately standardized redox measurement. A redox phenotype is considered a candidate predictive biomarker only when it is prospectively evaluated for treatment effect modification in a randomized trial; reproducible evidence of treatment effect modification and independent validation would be required before it could be considered a validated predictive biomarker. Candidate redox phenotypes include seminal ROS, ORP, lipid peroxidation, oxidative DNA damage (including sperm 8-OHdG), antioxidant capacity, mitochondrial dysfunction, and inflammatory markers, potentially integrated with clinical features such as obesity, smoking, varicocele, or metabolic disease (36).

No single redox measurement should be considered an actionable predictive biomarker solely because it correlates with infertility. Analytical validity, biological reproducibility, clinically relevant thresholds, and evidence of treatment effect modification are required.

8-OHdG provides a useful example of this evidentiary distinction. Primary human studies have reported higher sperm 8-OHdG in infertile men and associations with semen characteristics (34, 35), while studies examining reproductive outcomes have shown context-dependent findings across natural conception and assisted-reproduction settings (39–41). Its clinical interpretation is further constrained by assay heterogeneity and method-dependent thresholds (42, 43). Importantly, existing intervention studies have not established whether baseline sperm 8-OHdG modifies the effect of antioxidant treatment (34, 44). Thus, sperm 8-OHdG should currently be regarded as a clinically measurable candidate redox biomarker rather than a validated treatment-selection marker. Prospective trials should prespecify assay methodology and baseline 8-OHdG assessment and formally test a treatment by 8-OHdG interaction against clinically meaningful reproductive outcomes.

Efficacy testing should also be distinguished from predictive biomarker validation. A trial restricted to biomarker positive participants can test whether an intervention is efficacious within that selected population, but it cannot establish that treatment effects differ between biomarker positive and biomarker negative participants. For a binary biomarker, both categories should be represented, with treatment comparisons estimated within each category and a prespecified treatment by biomarker interaction. Even a significant interaction does not by itself establish a clinically meaningful benefit in the biomarker positive subgroup; subgroup treatment effects, confidence intervals, and clinically interpretable effect measures should be reported (38, 45, 46).

This distinction also explains why the existing literature cannot simply be “solved” by pooling more trials. If trials differ in biological inclusion criteria, interventions, doses, outcome definitions, and treatment contexts, a larger pooled sample may estimate an average effect more precisely while remaining unable to identify who benefits.

A parallel effort has proposed reframing idiopathic infertility around discrete redox endophenotypes hyper and hypo oxidative, DNA damage dominant, mitochondrial dysfunction, epigenetic redox, and inflammatory redox subtypes as a diagnostic reclassification strategy, with multicenter validation, omics integration, and biomarker driven algorithms proposed as future directions (36). That proposal and the framework developed here share a starting premise: a single “idiopathic” or “antioxidant responsive” label conceals mechanistically distinct populations. They differ in emphasis. The redox endophenotype model is primarily a diagnostic taxonomy; the framework proposed below is primarily a clinical trial architecture that specifies the prespecified interaction testing, target engagement criteria, and sample size requirements needed to determine whether any such taxonomy actually predicts treatment response. The two are complementary: a validated endophenotype classification would supply exactly the enrichment variable the trial framework below requires, and neither is sufficient without the other.

## A phenotype directed research framework

This framework extends, rather than replaces, existing diagnostic efforts such as the Male Oxidative Stress Infertility (MOSI) framework, which proposed oxidative stress driven infertility as a distinct, testable clinical entity (12). The contribution proposed here is a formal predictive biomarker validation pathway layered on top of that diagnostic foundation, specifying not only how a redox phenotype should be defined, but how a trial must be designed to determine whether identifying that phenotype changes which treatment a patient should receive. A phenotype directed framework should progress from clinical phenotype to a reproducibly characterized redox or mechanistic phenotype, mechanism matched intervention, target engagement, sperm biological response and clinical outcome. Candidate populations could include men with idiopathic oligoasthenoteratozoospermia, high sperm DNA fragmentation, obesity associated infertility, metabolic dysfunction, varicocele, or inflammatory phenotypes. These categories should not be presumed to be antioxidant responsive; they should be used to generate and prospectively test mechanistic hypotheses. The proposed framework is summarized in **Figure 1**.

**Figure 1:**
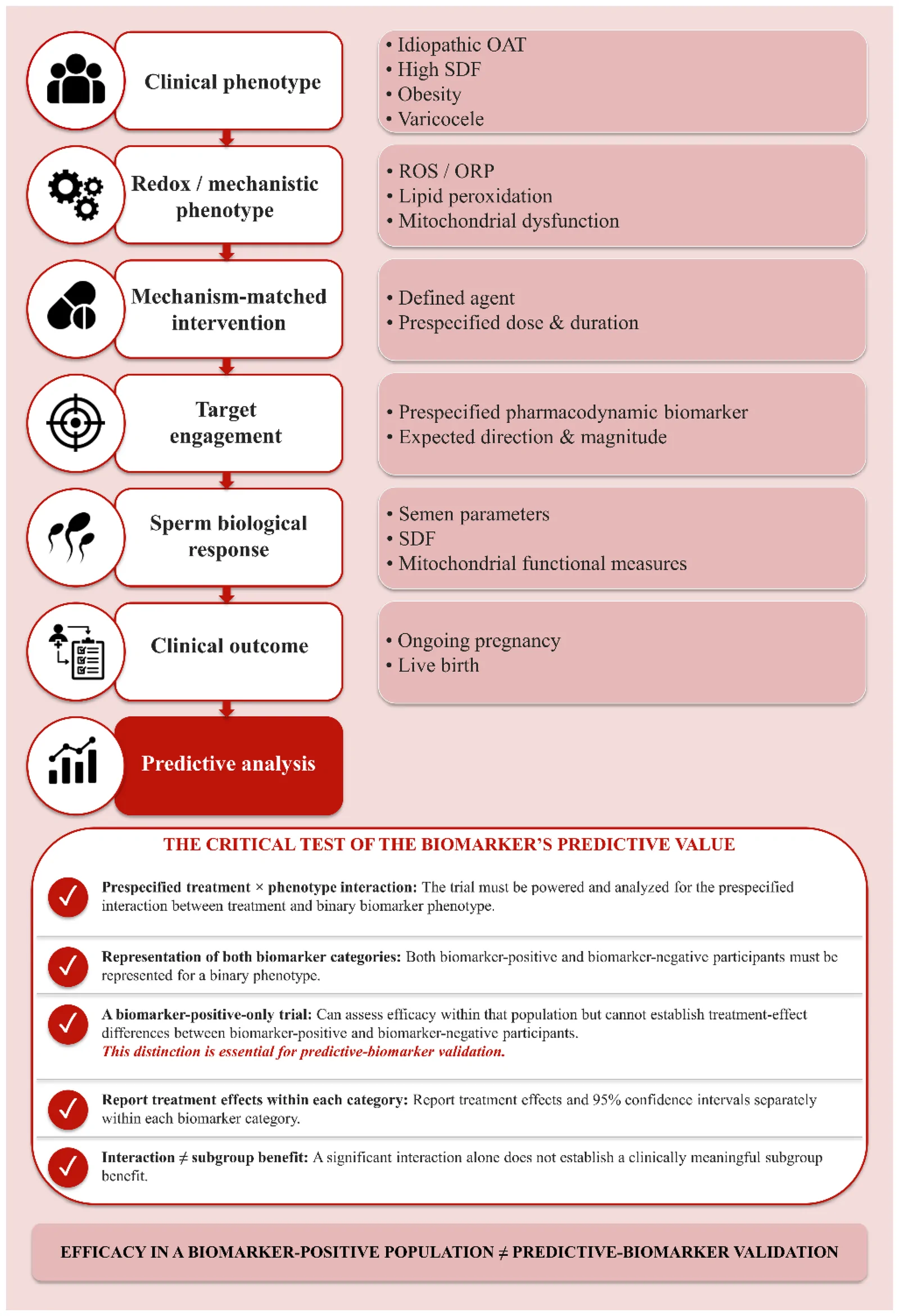
A phenotype directed framework for redox therapy. The proposed pathway moves from clinical phenotype to a reproducibly characterized redox phenotype, mechanism matched intervention, pharmacodynamic target engagement, sperm biological response, and clinically meaningful reproductive outcomes. The critical test of the biomarker’s predictive value is a prespecified treatment by redox phenotype interaction rather than a comparison of statistical significance within separate subgroups. For binary phenotypes, both biomarker categories should be represented, with subgroup treatment effects and 95% confidence intervals reported; a statistically significant interaction alone does not establish a clinically meaningful benefit in the selected subgroup. Candidate biomarkers shown are research variables, not validated clinical companion diagnostics. Oligoasthenoteratozoospermia, OAT; Reactive Oxygen Species, ROS; oxidation reduction potential, ORP; sperm DNA fragmentation, SDF.

## What should the next definitive trial look like?

First, eligibility should combine a clinically coherent infertility phenotype with a prespecified biological hypothesis. Second, the intervention should have a defined composition, dose, formulation, duration, and quality control specification. Third, placebo control should be used whenever feasible. Fourth, semen analyses should follow standardized WHO procedures, with repeat assessment when appropriate (2).

Fifth, the trial should be powered for a clinically meaningful reproductive endpoint. Ongoing pregnancy and live birth are preferable when feasible; semen parameters, sperm DNA fragmentation, ROS, and mitochondrial measures should be secondary or mechanistic endpoints (19). Sixth, the trial should prespecify the treatment by phenotype interaction and avoid *post hoc* “responder” claims based only on within subgroup P values (38). Continuous biomarkers should generally be modeled continuously when appropriate rather than dichotomized at arbitrary thresholds (45, 46).

Seventh, couple level factors must be incorporated. Pregnancy is not a male only endpoint; female partner age, ovarian reserve, infertility diagnosis, treatment modality, embryo characteristics, and assisted reproduction procedures can influence outcomes. Eighth, adherence and background supplement use should be monitored carefully. Finally, trials should report adverse events and potential harm. The lower ongoing pregnancy rate observed during the prespecified 4 to 6 month window, despite a neutral primary outcome over 6 months, also underscores the need for careful assessment of potential harms and temporal effects rather than assuming that nutritional supplementation is biologically neutral (23).

## What the evidence does and does not show

The current evidence does not support routine empirical antioxidant supplementation as a universally effective treatment for male infertility (19–21, 23). The MOXI and SUMMER trials provide particularly important evidence against broad multicomponent supplementation in the populations and formulations studied (20, 23). At the same time, the evidence does not justify the stronger statement that all redox modulating interventions are ineffective in all men. Individual agent studies and systematic reviews continue to report biological and semen quality signals for some interventions (24–29), while the certainty of evidence on pregnancy and live birth remains low or very low (19).

The most defensible conclusion is therefore uncertainty with direction. The field should stop treating “antioxidant” as a sufficient therapeutic definition and determine whether redox dysfunction can be measured reproducibly, whether it is causally relevant, and whether it can identify men who benefit from a defined intervention.

The phenotype directed model also creates a more informative hierarchy of evidence. At the discovery stage, investigators can use broad multi-omic or functional profiling to identify candidate redox states. At the validation stage, a smaller set of assays should be tested for reproducibility, biological stability, and association with clinically relevant outcomes. At the prediction stage, the biomarker must be evaluated prospectively for treatment effect modification. This sequence prevents a common translational error: selecting a biomarker after observing that a treatment worked better in one subgroup and then presenting that subgroup as if it had been prospectively identified.

Statistically, the key statistical test of a candidate predictive biomarker is treatment effect modification. For a binary reproductive outcome such as ongoing pregnancy or live birth, this can be evaluated with a prespecified regression model containing randomized treatment assignment, the prespecified baseline redox phenotype, their treatment by phenotype interaction, and a limited set of prespecified baseline covariates. For example, for a binary outcome, an illustrative logistic regression model is logit[P(Y = 1)] = β_0_ + β_1_T + β_2_P + β_3_(T×P) + Σβ_k_C_k_, where Y is the binary outcome, T is treatment assignment, P is the prespecified baseline redox phenotype, and β_3_ tests whether the treatment effect differs according to phenotype. For a prespecified time to live birth outcome, an analogous treatment by phenotype interaction could be incorporated into an appropriate survival model, such as a Cox proportional hazards model when its assumptions are satisfied; for continuous sperm outcomes, an appropriate linear or mixed effects model could be used. Thus, the specific model depends on the endpoint, but the underlying principle is invariant: predictive treatment effect heterogeneity should be evaluated through a formal interaction between treatment and the prespecified redox phenotype rather than inferred from separate within subgroup P values (45, 46).

Sample size planning must therefore account for interaction testing, which generally requires substantially more participants than a trial designed only to detect a main treatment effect. A future trial that proposes to identify a responder phenotype but is powered only for the overall treatment comparison risks repeating the evidentiary problem it seeks to solve. If multiple candidate biomarkers are tested, multiplicity should be controlled or the analysis should clearly distinguish confirmatory from exploratory hypotheses (45, 47). Independent replication is particularly important because treatment effect heterogeneity is statistically fragile and can arise by chance.

Target engagement should be incorporated prospectively. If a compound is hypothesized to reduce oxidative damage, the trial should specify the expected direction and magnitude of change in a prespecified pharmacodynamic marker and determine whether that change occurs preferentially in the prespecified target phenotype. A trial in which the intervention fails to alter the biomarker cannot be interpreted as a definitive test of the pathway unless adequate exposure and assay performance have been demonstrated. Conversely, successful target engagement followed by a neutral reproductive outcome would be mechanistically informative: it would suggest that the targeted redox pathway may not be rate limiting for fertility in the studied population, or that downstream biological redundancy prevents clinical translation.

## Conclusions

The antioxidant literature in male infertility represents a striking mismatch between mechanistic plausibility and clinical convergence. Decades of laboratory research and numerous RCTs have produced signals across semen quality, sperm function, oxidative biomarkers, and DNA integrity, yet the evidence has not established a reproducible improvement in clinically meaningful reproductive outcomes (19–21, 23).

The 2025 WHO guideline therefore responds to this uncertainty by making no recommendation rather than an unsupported endorsement or universal rejection of antioxidant supplementation (14–16). The SUMMER trial found no improvement in ongoing pregnancy with the tested formulation in the enrolled population (23). Whether a reproducible redox phenotype can prospectively identify differential antioxidant treatment benefit through a prespecified treatment by phenotype interaction on clinically meaningful reproductive outcomes remains unestablished.

The next generation of trials should therefore move beyond the binary question of whether antioxidants work and instead determine whether a reproducibly measurable, biologically relevant redox phenotype identifies patients who derive clinically meaningful benefit from a defined intervention. Such trials should prespecify phenotype classification, demonstrate target engagement, test treatment by phenotype interaction, control multiplicity, and prioritize ongoing pregnancy and live birth as clinically meaningful outcomes. This framework does not assume that antioxidants work. It tests the more informative proposition that a measurable, biologically relevant redox phenotype may identify men in whom a defined redox modulating intervention improves reproductive potential. Until such evidence is available, antioxidant supplementation should remain a biologically plausible but heterogeneous intervention whose clinical value cannot be assumed across unselected populations.
